# India Rubber

**Published:** 1879-12

**Authors:** 


### INDIA-RUBBER.

The British Consul at Panama reports that India-rubber has almost ceased to be an article of export from the isthmus, mainly in consequence of the great difficulty and expense of getting at the trees in the remote districts of the interior. Those nearer the coast have been destroyed by the wasteful system pursued by the natives in cutting down the trees to procure the sap.—*Scientific American.*

